# PathExNET: A tool for extracting pathway expression networks from gene expression statistics

**DOI:** 10.1016/j.csbj.2021.07.033

**Published:** 2021-07-29

**Authors:** George Minadakis, Alfonso Muñoz-Pomer Fuentes, George Tsouloupas, Irene Papatheodorou, George M. Spyrou

**Affiliations:** a Bioinformatics Department, The Cyprus Institute of Neurology & Genetics, 6 Iroon Avenue, 2371 Ayios Dometios, Nicosia, Cyprus; b The Cyprus School of Molecular Medicine, 6 Iroon Avenue, 2371 Ayios Dometios, Nicosia, Cyprus; c European Molecular Biology Laboratory, European Bioinformatics Institute, EMBL-EBI, Hinxton, UK; d HPC Facility, The Cyprus Institute, 20 Konstantinou Kavafi Street, 2121, Aglantzia, Nicosia, Cyprus

**Keywords:** Pathway expression networks, Differential expression analysis, Pathway analysis

## Abstract

A fundamental issue related to the understanding of the molecular mechanisms, is the way in which common pathways act across different biological experiments related to complex diseases. Using network-based approaches, this work aims to provide a numeric characterization of pathways across different biological experiments, in the prospect to create unique footprints that may characterise a specific disease under study at a pathway network level. In this line we propose PathExNET, a web service that allows the creation of pathway-to-pathway expression networks that hold the over- and under expression information obtained from differential gene expression analyses. The unique numeric characterization of pathway expression status related to a specific biological experiment (or disease), as well as the creation of diverse combination of pathway networks generated by PathExNET, is expected to provide a concrete contribution towards the individualization of disease, and further lead to a more precise personalised medicine and management of treatment.

PathExNET is available at: https://bioinformatics.cing.ac.cy/PathExNET and at https://pathexnet.cing-big.hpcf.cyi.ac.cy/

## 1 Introduction

Analysis of differential gene expression profiles often generates top-scored gene-sets on which pathway-based enrichment analysis is routinely performed, leading to a statistically significant list of pathways, that may be related to the underlying biology of the condition being studied [7], [28], [51]. The challenge through these types of analyses is to find specific pathways affected by a group of related genes, namely pathways perturbed by differentially expressed genes. Although such tools may reveal significant top-scored pathways, the pathway complexity and the varying characteristics of genes do not easily allow to optimally relate these pathways to a specific biological condition being studied [18]. Despite the magnificent efforts of differential expression analysis pipelines, generating unbiased results is still a challenge, while common aetiologies of such failures usually vary between issues related to the experimental setup and difficulties in customization of the statistical analysis tools [9], [26]. Scoring and filtering of differentially expressed data results to a loss of a large amount of important yet not statistically significant genes, where despite their weak statistical significance, their contribution into the biology of the condition being studied remains a relatively unexplored scientific issue. Another crucial confusing issue scientists usually face through enrichment analysis, is a quite significant list of top-scored pathways that are common across a variety of diverse diseases [42], [46]. For example, the pathway of “apoptosis“ is a very common pathway that appears very often in several studies, while less generic pathways such as “N-glycosylation” or “N-glycan biosynthesis” have been also associated with a series of congenital disorders [13]. However a pathway’s association across different diseases, by no means suggests in biochemical and/or biological terms, that a specific pathway contributes in the same way to all types of diseases in which may be identified. Indeed, biological pathways can be considered as topological networks formed by sets of genes or molecules that interact through chemical reactions, molecule modifications or signal transduction [5], [48]. Thus their significance should be a result that derives from the integration of both gene-set analysis and topology information [35], [48]. In this line, network-based approaches have proved to be a promising Systems Bioinformatics framework of analysis, both at gene and pathway analysis level [22], [38], [53]. In the prospect to develop of additional tools able to enrich the outcome of the routinely performed pipelines used for this type of research, the present work aims to explore whether the overall differential expression information of the genes included in a specific pathway is adequate to give a different characterization for the same pathway across different diseases. Using network-based approaches, in this work we present a methodology and a related web-service for the numeric characterization of pathways across different differential expression datasets, able to provide unique pathway network footprints, which in turn may represent a specific biological condition (and/or disease) under study. In this line, we propose PathExNET, a web service that allows the creation of pathway expression networks that hold the over- and under-expression information obtained from differential gene expression analyses. PathExNET holds a large database of reference pathway-to-pathway networks, which have been developed through the freely available information included in the KEGG, Reactome and Wiki Pathways database repositories. Users can upload their differential gene expression statistical analysis, followed with pathways and/or genes of interest, and further chose a scoring methodology to create and explore the derived pathway-to-pathway expression networks. In order to provide a concrete set of well-evaluated differential gene expression statistical analyses and to further increase the data-availability and easy data access of PathExNET, an additional tool has been rooted in PathExNET framework that allows to search and directly import pre-processed statistic files from the Expression Atlas (EA) (https://www.ebi.ac.uk/gxa) data resource of the European Bioinformatics Institute (EMBL-EBI) (https://www.ebi.ac.uk).

## 2 Software description & methods

By definition, the term Pathway Expression Networks (PENs) employed in this work refers to pathway-to-pathway networks, where: (a) the nodes are pathways, the node size and the node colour represent a specific parameter that characterizes the level of over- and under-expression statistical information of genes included in a specific pathway, and (b) the edge-weight represents the number of common genes between two pathways. PENs draw from the log-fold-change (logFC) parameter obtained through the Differential Expression Analysis (DEA) of genes. The pathway characteristic parameter is obtained by means of four diverse methodologies employed in this work. In the following we describe in detail the main components and methodologies used for the implementation of the proposed tool.

### 2.1 Overall design and software availability

PathExNET comes with a frontend web interface that consists of the mainframe and a help page, written in HTML, PHP and JavaScript language environments. The mainframe provides 2 individual steps designed to guide the user until the end of the workflow process. The backend of PathExNET has been written in R environment, where several functionalities have been parallelised to achieve fast performance. Evaluation, testing and understanding of PathExNET functionalities can be easily performed by means of several available example datasets provided through the web interface. The proposed tool is available online at the webpage of the Bioinformatics Department, at the Cyprus Institute of Neurology and Genetics (CING) (http://bioinformatics.cing.ac.cy). PathExNET is served by a Docker space at the CYTERA High Performance Computer Facility of the Cyprus Institute (https://hpcf.cyi.ac.cy). PathExNET further uses parallel processing scripts to handle and pre-process large file sizes that make the use of the “doParallel” R package [4].

### 2.2 The pathway reference network repository

The pathway-to-pathway network information draws from a web-service that holds a large database of reference pathway networks, which have been developed through the freely available information included in the KEGG [41], Reactome [11] and Wiki Pathways [25] database repositories. Herein, the functional relation between two pathways that forms an edge in a network, is considered when a specific pathway involves or is being involved in to another pathway accordingly. In effect, this type of information which is mainly obtained from the available XML maps of the above mentioned repositories, can form an undirected-unweighted pathway-to-pathway network. In this line of thought, a large number of pathway XML maps were obtained for all the organisms included in the three above mentioned repositories, and all the available data related to the functional connections that exist between all the available pathways were retrieved. Specifically, we obtained 177 organisms from KEGG, 16 from Reactome and 38 from Wiki Pathways repositories, accordingly. The output of this data mining process was further used to construct in total 231 undirected pathway reference networks, stored in a data repository. Further information rooted in these reference pathway-to-pathway networks, involves the number of common genes between two pathways that forms the edge-weight of these networks, and the number of total genes included in a pathway that forms the node size. The underlying networks are regularly updated, constructing the main pathway repository for the services and methodologies that PathExNET draws from. It should be noticed that an initial version of this reference network repository supporting only 16 organisms from KEGG and Reactome repositories, has been recently used in PathwayConnector [34], [35], with noteworthy results to pathways related to Alzheimer’s Disease (AD) [53], to Huntington’s disease (HD) and Spastic Ataxia (SA) [23], as well as to a recent study on Breast Cancer [16].

### 2.3 The expression Atlas searching and importing tool

The Expression Atlas (EA) is a database repository that provides information about gene and protein expression in different species and contexts, namely: tissue, developmental stage, and disease or cell type. The EA web service is hosted at the European Bioinformatics Institute (EMBL-EBI) (https://www.ebi.ac.uk). EA holds a large set of publicly available and controlled access datasets that at the time of writing derive from over 4,000 studies across 65 different species, including over 900 studies from plants. These datasets have been curated and re-analysed using standardized, open source pipelines and have been made available along with the analyses data for queries, download and visualization [40]. EA incorporates baseline expression profiles of tissues from Human Protein Atlas, GTEx and FANTOM5, as well as of cancer cell lines from ENCODE, CCLE and Genentech projects. Through the last update EA incorporates data from large-scale RNA sequencing studies including Blueprint, PCAWG, ENCODE, GTEx and HipSci. In order to provide a concrete set of well-evaluated DEA data files, a productive collaboration with EA team led to the development of an additional search tool, rooted in PathExNET framework. The underlying tool allows users to search and directly import into PathExNET pre-processed DEA files. Users can search by means of specific EA experiment accession, organism, and experiment type, or alternatively perform free text keyword search.

### 2.4 Creating pathway expression networks

There are three input combinations where users can provide to create PENs: (a) DEA file accompanied with list of pathways of interest, (b) DEA file accompanied with list of genes of interest, and (c) DEA file accompanied with a list of pathways and a list of genes of interest. A significant differentiation in this approach is that PENs use all the genes included in the experiment, thus the DEA files should be used unfiltered without performing any specific threshold for reducing their size. The DEA file should at least include the gene-symbol and the log-fold-change value for each gene included in the file, while the p-value field is optional. These parameters should be strictly named as: “*Gene.symbol*”, “*logFC*”, and “*P.Value*”, accordingly. Pathways and genes of interest may derive from any type of omics data analysis that leads to significant pathways and genes accordingly. The proposed methodology for the creation of PENs reads as follows. For a specific pathway of interest, our methodology first finds all the genes included in the pathway. This type of information is obtained from the pathway reference network repository described in the previous section, which holds all the genes involved in each pathway. These genes are further matched with those that derive from the DEA files, where the logFC value is attached for each one of these genes. Synonyms of gene symbols are also considered in this process in the prospect to reduce the number genes that may be missed through this type of matching. The next step of our method involves the assignment of a specific numeric value to the pathway of interest by means of the following equations:

The sumFC value is obtained by calculating the sum of all the log-fold-change values included in the specific pathway, as follows:

(1) $$\mathrm{sumFC}=\sum_{i=1}^{N}log\left(FC\right)$$

where Nrefers to the total number of genes included in the pathway, and $\log\left(FC\right)$ is the logarithmic representation of the fold-change (FC) value. When this overall score is above zero, the specific pathway of interest is mostly considered as an over-expressed pathway. On the contrary for negative values ofsumFC the pathway is mostly considered as an under-expressed pathway. However this approach may provide biased results since the underlying score does not take into account the balance between the number of over and under expressed genes in a sample. For example, four genes with logFC values of −0.2, −0.3, −0.1 and 0.8, would give a sumFC=0.2, suggesting an over-expressed network, against the fact that the sample includes more under-expressed genes that over-expressed ones. To handle with this limitation we proceed with two additional equations that lead to a combined score. These read as follows:

The rateFC value is the fraction of the number of over-expressed genes divided by the number of total genes included in the pathway, as follows:

(2) $$\mathrm{rateFC}=\frac{\#ofover-expressedgenes}{\#ofgenes}$$

ForrateFC≥0.5, the specific pathway of interest is mostly considered as an over-expressed pathway. On the contrary for rateFC≤0.5the pathway is considered as an under-expressed pathway.

The normMeanFC value is obtained by calculating the weighted mean of the normalised histogram of the log-fold-change values. Specifically, for a given vector of log-fold-change values $V_{\mathrm{LFC}}=v_{1},v_{2},\cdots v_{n}$ we first apply a normalisation function in order to restrict the values within the range of$V_{\mathrm{LFC}}\in$.

(3) $$V_{\mathrm{norm}}=\left(V_{\mathrm{LFC}}-min\left(V\_LFC\right)\right)/\left(max\left(V_{\mathrm{LFC}}\right)-min\left(V\_LFC\right)\right)$$

Then the histogram of the normalised vector$V_{\mathrm{norm}}$, is calculated using a bin of 0.01, which results to N=100 ranges ($x=x_{1},x_{2},\cdots x_{100}$), represented by their frequencies$F(x_{i})$, which in turn are used to calculate the weight vector$W\left(x_{i}\right)=\frac{F\left(x_{i}\right)}{N}$. The weighted mean is then obtained by the following equation:

(4) $$\mathrm{normMeanFC}=\frac{\sum_{i=1}^{N}W\left(x_{i}\right)x_{i}}{\sum_{i=1}^{N}W\left(x_{i}\right)}$$

Eq. (4) suggests that for a normal distribution of logFC values, those with a larger weight contribute more to the weighted mean than those with a smaller weight. In effect a numeric mean characterisation of a pathway will be based on most frequent values that exist in the vector. Herein, fornormMeanFC≥0.5, the specific pathway of interest is mostly considered as an over-expressed pathway. For normMeanFC<0.5,the pathway is considered as an under-expressed pathway. The underlying metric aims to slightly fix the ambiguity in the sumFC balancing by estimating the normalised weight of the distribution of the logFC values included in the sample.

**The combinedFC value** is a combination of equations (2), (4) as follows:

(5) combinedFC=rateFC+normMeanFC

Typically forcombinedFC≥1.0, the specific pathway of interest is mostly considered as an over-expressed pathway. For combinedFC<1.0,the pathway is considered as an under-expressed pathway.

In order to estimate a score for the overall pathway expression network we calculate the overall expression ratio which is defined as the fraction of:

(6) $$R_{\mathrm{NET}}=\frac{\#ofover-expressedpathways}{\#ofpathways}$$

For values of$R_{\mathrm{NET}}\geq 0.5$, the network is considered as an over-expressed network, while for values$R_{\mathrm{NET}}<0.5$, the network is considered as an under-expressed network, respectively.

Herein, we clarify that there is not an optimal theory that clearly defines where is the transition line between over- and under-expression of gene sets. The most widely used methods that use the log-fold-change value, mainly examine how the logFCvalue is different from zero, without suggesting any biologically objective truth [33]. Thus the transitions of 0.5 and 1.0 used in the above equations have been arbitrary selected, assuming that the logFC values included in a gene expression dataset, which has been transformed and normalised successfully, follow a typical normal Gaussian-like distribution around zero.

### 2.5 Performing enrichment analysis

In order to provide an indicative information that shows whether the selected by the user pathways are also significant in terms of enrichment analysis, we used the “gprofile2” R package which has been also suggested as a main analysis tool by the ELIXIR consortium [29]. Specifically, when users provide lists of genes, the tool performs pathway enrichment analysis by using these genes. The enrichment score (namely the p-value) obtained for each selected pathway, is now provided on the visualised networks, especially when the user puts the mouse cursor over a specific node. To further handle the large network problem we rooted into the tool the possibility to select the maximum number of top-scored pathways to be visualised in the network. The ranking is based on the p-value score obtained from enrichment analysis of the given gene-set. Herein the limitation we find in this approach is that insignificant lists of pathways provided by the user may not be included in the enrichment result. In that case the enrichment score is simply NA for those pathways.

### 2.6 Providing gene regulatory information

Another significant issue in studying the expressional behaviour of pathways is the regulatory information in between the genes included in a specific pathway. Thus in order to provide such information through PathExNET framework, we further created a database repository that includes regulatory information in between genes, obtained from both the *Transcriptional Regulatory Relationships Unraveled by Sentence-based Text mining* (TRRUST) [20], and the *SIGnaling Network Open Resource* (SIGNOR) [31] repositories. The underlying repository includes regulation information about 95,086 pairs of genes. However, the specific information, although significant, remains limited since it is available for only three species: *Homo Sapiens*, *Mus Musculus*, and *Rattus Novergicus*, accordingly. Depending on the user’s input, PathExNET automatically examines the genes of interest and further provides the regulatory information to where is available, in a single table.

### 2.7 Exporting network statistics

The mathematical content of complex networks in biological systems has become a benchmark approach towards identifying biomarkers, understanding their dynamics, their biological status and related biological mechanisms involved. In order to provide more statistics related to the complex nature of the proposed pathway expression networks, PathExNET further provides additional statistics, for network manipulation [8]. These indicatively include measures of median, mean and maximum values of: betweeness-centrality, degree distribution, closeness, and clustering coefficient. This attempt aims to create a concrete web-framework of analysis adequate to provide a multilevel information content, sufficient for further investigation and understanding of pathway networks.

## 3 Demonstration of PathExNET capabilities

It should be stressed that the concept of PathExNET is not to serve as enrichment tool but as a post analysis tool that facilitates an estimation and the subsequent visualization of the collective over/under expression of the selected pathways’ gene members. As opposed to traditional gene enrichment tools and methodologies [30], [37], [45], PathExNET allows users to create pathway expression networks in order to evaluate specific biological conditions where pathways or genes of interest are not necessary significant, namely a high-score result of a ranking methodology. Thus the equations provided in PathExNET have been designed in a simplified manner in order to be independent of any gene or pathway rankings. In the following subsections we present two different case studies in order to support this argument and to show how same clusters of pathways behave across different gene expression datasets.

### 3.1 A case study on SARS-CoV-2 experimental data

**Fig. 1:**
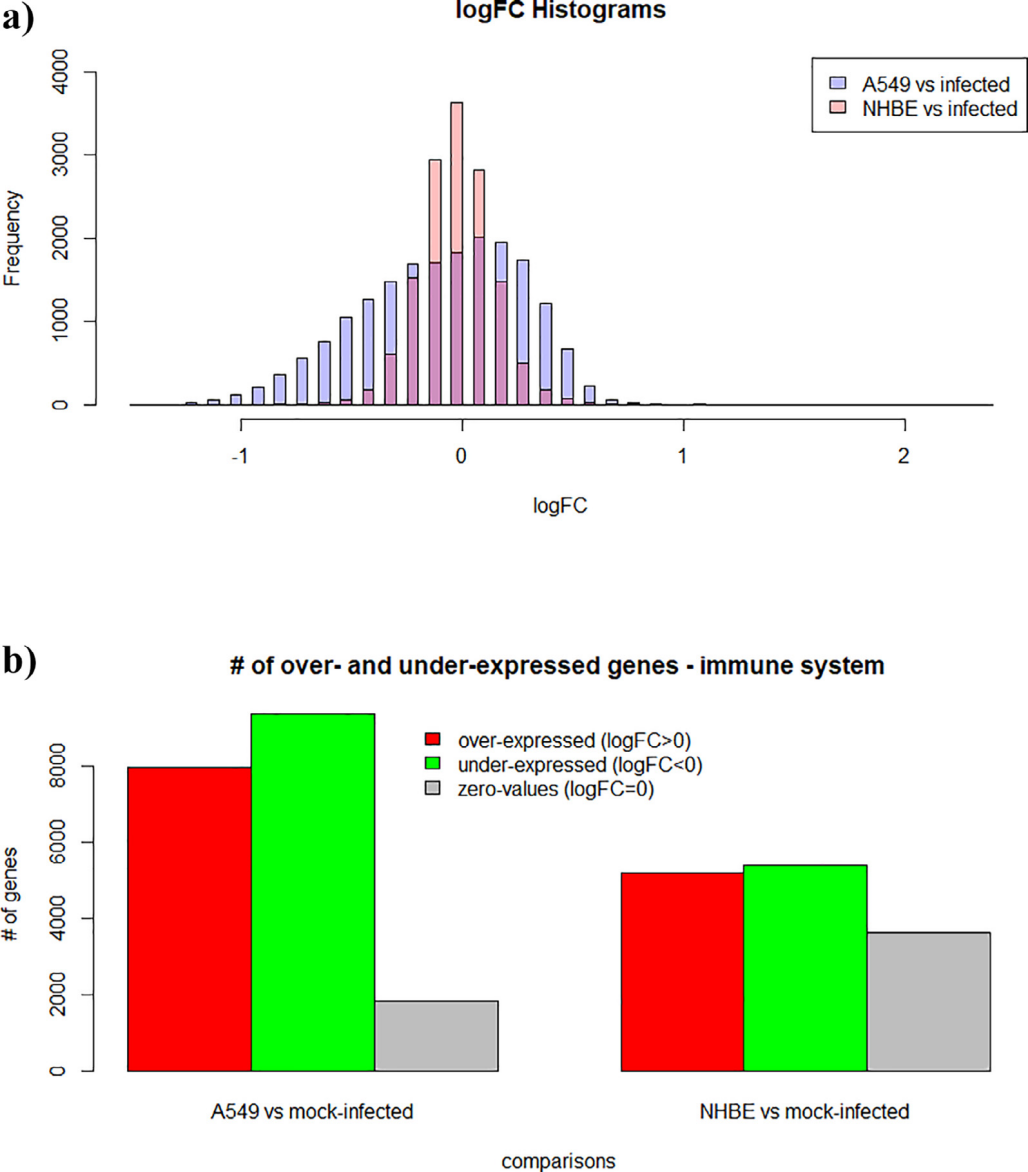
(a) Depicts the frequency distribution of logFC values obtained from the differential expression analysis of A549 and NHBE comparisons, (b) depicts the number of over- and under-expressed genes per comparison, estimated by means of the logFC parameter**.**

A common biological perspective for an effective treatment against COVID-19 and its causative virus, SARS-CoV-2, is the deciphering of the involved host pathways, as well as the related transmission and replication mechanisms [6], [19], [44]. In this line of thought, our approach here focuses on the examination of the over-expressed and under-expressed gene content of specific categories of pathway networks related to COVID-19. On this ground, we are using PathExNET to analyze a recently introduced high throughput sequencing expression dataset, related to the transcriptional response of human lung epithelial cells to SARS-CoV-2 infection [3]. The dataset includes expression profiles of two independent biological triplicates of: (i) normal human bronchial epithelial (NHBE) cells and (ii) transformed lung alveolar (A549) cells, which were both mock treated or infected with SARS-CoV-2 (USA-WA1/2020). The underlined subset has been analysed by the team of EA, who performed differential expression analysis, available on EA repository at https://www.ebi.ac.uk/gxa/experiments/E-GEOD-147507. Herein, we used the PathExNET EA tool to download the analysis performed by EA, namely the unfiltered statistics required for the creation of the pathway expression networks proposed in this work. Fig. 1a depicts the frequency distribution of the logFC parameter included in these statistics, showing that both samples are well-distributed around the zero point. In addition, Fig. 1b depicts the over- and under-expressed information estimated by means of the logFC parameter. Both samples seem to exhibit the same behaviour between the over- and under- expressed genes, with estimated ratios $R_{A549}=0.46$, and $R_{NHBE}=0.49$, accordingly, where the ratio R refers to the fraction of the number of over-expressed genes, to the total number of genes included in the sample.

**Fig. 2:**
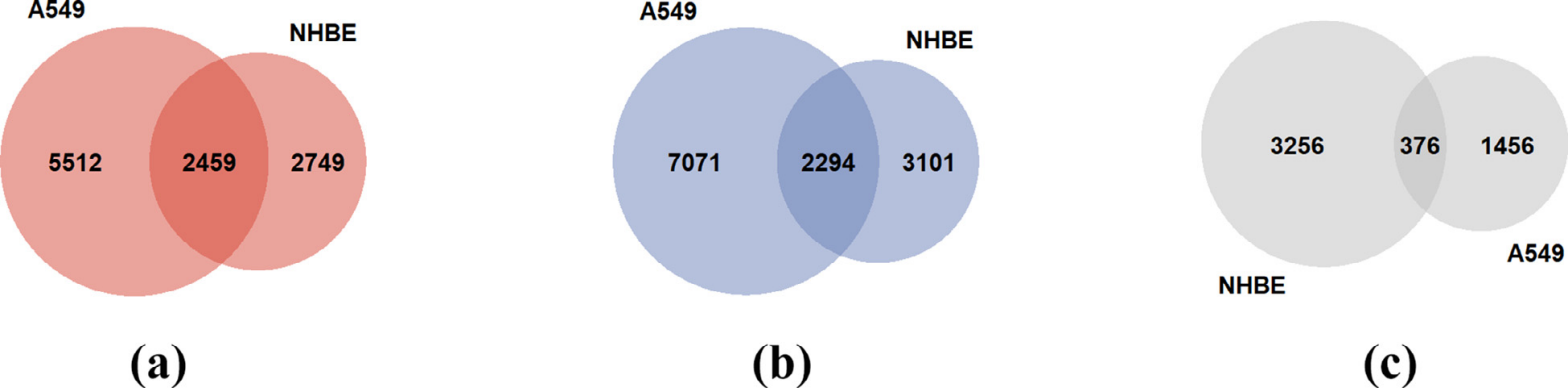
Venn diagrams representing the datasets overlapping for: (a) their over-expressed genes, (b) their under-expressed genes, (c) their genes with zero logFC values, included in the two cell lines under study.

Furthermore, Fig. 2 depicts the *Venn* diagrams that represent the overlap between the two samples regarding (a) their over-expressed genes, (b) their under-expressed genes, and (c) their genes with zero logFC values, respectively. It is observed that both samples share almost the same amount of common over-expressed and under-expressed genes, which in effect secures that the obtained pathway expression networks will be a result of a well-balanced common genes included in these pathways.

**Fig. 3:**
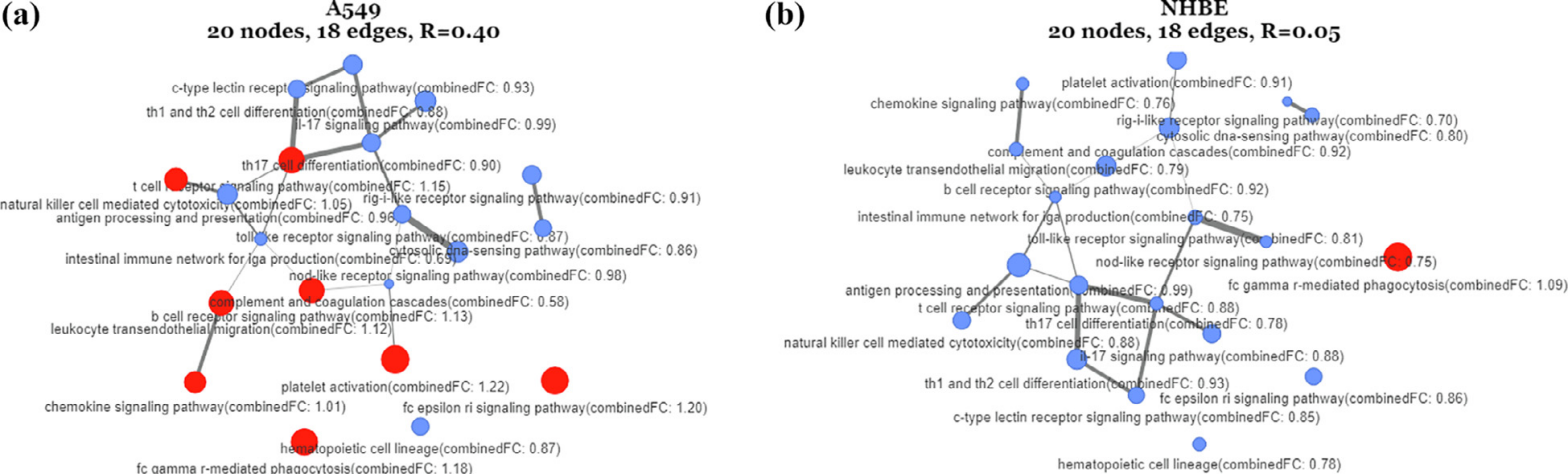
(a, b) Pathway expression networks of 20 KEGG pathways related to immune system. The node values have been estimated by means of the combinedFC parameter. The logFC values have been obtained from the A549 and NHBE datasets**.** The red nodes refer to the pathways with high content of over-expressed genes (combinedFC≥1.0), while the blue nodes refer to the pathways with high content of under-expressed genes (combinedFC<1.0). (For interpretation of the references to color in this figure legend, the reader is referred to the web version of this article.)

It has been stated that for COVID-19, as well as for all infectious diseases, the host immune system, is a major component [2], [14], towards understanding the host response on the infection. In this line, 20 pathways related to the immune system, were obtained from the KEGG pathway repository, in order to be analyzed by means of the PathExNET methodology. The compiled pathway list, was further used to create pathway-to-pathway expression networks, in combination with the differential expression statistics obtained from EA, by means of the PathExNET methodology proposed in this work. Specifically, Fig. 3a depicts a pathway expression network that includes the 20 candidate KEGG pathways, where the node (pathway) values have been estimated by means of the combinedFCscore (see Eq. (5)) described in previous section. The logFC values have been obtained from the A549 analysed dataset. The edge-weights refer to the number of common genes between the two pathways that form the edge. Fig. 3b depicts the same analysis for the NHBE differential expression dataset accordingly. As opposed to the colour scale provided by the web tool, here in order to show the expression difference in between these two networks, an arbitrary transition threshold was selected while the colour scale used includes only two colours. Specifically, the red-coloured pathways refer to the over-expressed pathways (combinedFC≥1.0), while the blue ones are the under-expressed ones (combinedFC<1.0). The overall network expression ratio (see Eq. (6)) was found$R_{\mathrm{NET}}=0.40$ for the A549 gene-set, and $R_{\mathrm{NET}}=0.05$ for the NHBE gene-set, suggesting that the first is considered as an enriched network of pathways with higher content in over-expressed genes in contrast to the second one. In consequence, the latter methodology suggests that pathway networks obtained from the given set of gene expressions, may have a unique identity in terms of the proposed scores, which in effect may contribute to a unique characterization of the specific biological condition under study. It should be stressed that the specific analysis draws from a simple biological concept that aims to examine the expression status of all the immune system pathway networks, subjected to SARS-COV-2 infection.

**Fig. 4:**
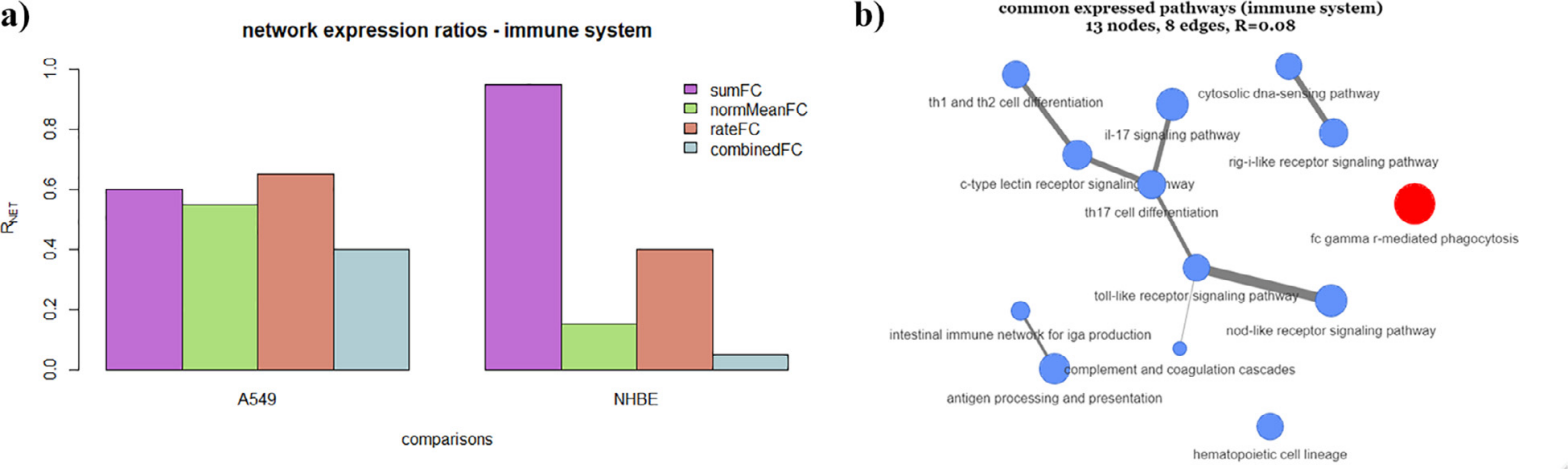
(a) Estimated network expression ratios$R_{\mathrm{NET}}$ obtained from A549 and NHBE datasets. The different colors refer to different equations used for the numeric characterization of each pathway. (b) 13 of 20 commonly expressed pathways obtained from the analysis of A549 and NHBE datasets.

Expanding on this type of analysis, the latter analysis was performed for all the proposed scores derived by the described in previous section, for each of the analysed datasets under study. Specifically, Fig. 4a depicts the estimated network expression ratios$R_{\mathrm{NET}}$of the two groups A549 and NHBE, for the different scores used for the numeric characterization of the 20 KEGG pathways related to immune system. It is observed that the network expression ratios that derive from normMeanFC, rateFC and combinedFC equations, are higher in the case of A549 analysed dataset. On the contrary the network expression ratios that derive from the sumFC parameter, exhibit different behaviour, as expected, due to the balancing issues mentioned in previous section. In effect, the above results lead to the assumption that the immune system pathway networks, are more likely to exhibit higher expression ratio in the case of the transformed lung alveolar cells (A549) that have been infected by SARS-COV-2, rather than in the case of human bronchial epithelial cells. In order to further support the latter assumption as well as the validity of this finding, we further focus on a common biological factor that applies to the cell-line context. Different cell-line experiments usually target on different candidate genes that may trigger a specific biological condition. In effect this leads to the suspicion that the over- and under-expressed behaviour observed in the above pathway expression networks may be a result of the pathways that involve these genes. Commonly expressed pathways are more likely to involve genes that do not significantly change the network expression ratio. In this line of thought, we further performed analysis on pathway commonalities across the two different biological conditions under study. Specifically, based on the expression ratio$R_{\mathrm{NET}}$, estimated by means of the combinedFC score, we further identified which pathways are commonly expressed across the two analysed datasets under study. This process aims to identify whether the higher over-expressed behaviour of the immune system pathways observed in the case of A549 pathway network, is triggered (or-not) from the common expressed pathways involved in-between the two biological conditions under study. In this context, Fig. 4b shows the commonly expressed pathways related to the immune system, obtained from the analysis of A549 and NHBE comparisons. Here, we observe that the expression networks obtained by means of the commonly expressed biological mechanisms involved in between the A549 and NHBE experiments, exhibit relative low-expression ratio $R_{\mathrm{NET}}=0.08$.

**Fig. 5:**
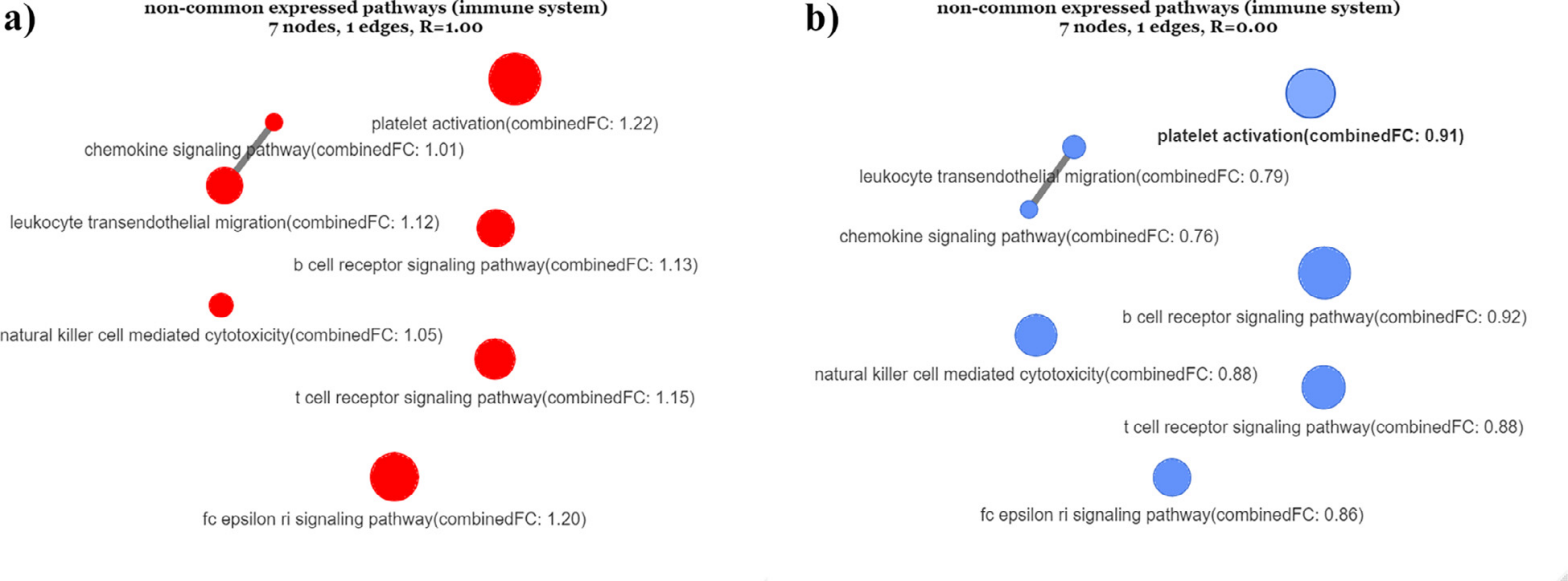
Pathway expression networks of 7 non-common expressed KEGG pathways related to immune system, where the logFC values have been obtained from (a) the A549 and (b) the NHBE datasets, accordingly. The node values have been estimated by means of the combinedFCparameter**.** The red nodes refer to the over-expressed pathways (combinedFC≥1.0), while the blue nodes refer to the under-expressed ones (combinedFC<1.0). (For interpretation of the references to color in this figure legend, the reader is referred to the web version of this article.)

In analogous manner, Fig. 5 depicts those pathways that are not commonly expressed in-between the two cell-line experiments. Here it is clearly observed that the pathways enumerated by means of the logFC values included in the A549 analysed dataset, are all over expressed in contrast to the NHBE dataset, where the obtained expression ratios found $R_{\mathrm{NET}}=1.00$ for the A549 gene-set, and $R_{\mathrm{NET}}=0.00$ for the NHBE gene-set, accordingly. The above results clearly show that non-commonalities in between the two experiments exhibit a highly expressed network in the case of A549 gene-set, while a low-expressed one is observed in the case of NHBE gene-set, accordingly.

To further support the validity of the above results, we indicatively performed a literature search for the 9 over expressed immune system pathways depicted in Fig. 3. Specifically, the *“fc gamma r-mediated phagocytosis”* and *“fc epsilon ri signaling pathway”* pathways which were found to be over-expressed in both A549 and NHBE expression networks, have been discussed in relevant studies that relate its involvement with antiviral immune responses to SARS-COV-2 infections [39], [49], [50]. Another pathway which has been found to be over expressed in the A549 experiment is the “*chemokine signaling pathway”* mentioned in [3], who performed the experiments this work draws from. The authors observed a consistent chemokine signature in the A549 dataset, despite the reduced IFN-I and IFN-III response to SARS-CoV-2. Two additional over-expressed signaling pathways, namely the b-cell and t-cell receptors, have also been recently linked to SARS-COV-2 in [1], [17], [52]. Additional studies highlight the necessity of comprehensive studies on “*natural killer cell mediated cytotoxicity*” in COVID-19 patients [21], [32]. The “*leukocyte transendothelial migration*” has been also associated with ACE2 expression [12], and has also derived from gene enrichment analyses [47]. Finally, the “*platelet activation”* mechanism has been found to be promoted by the TLR9 receptor, through the “*Interleukin-1 receptor-associated kinase 1 (IRAK1)”* and “*protein kinase B (Akt/PKB)”* pathways [15].

### 3.2 A case study on Colorectal Cancer experimental data

In this example we focus on a well-designed dataset that includes RNA-seq data of 54 samples, obtained from 18 patients with primary Colorectal Cancer and liver metastasis [27]. The dataset is available at the EA repository (https://www.ebi.ac.uk/gxa/experiments/E-GEOD-50760) while the experimental design involves two comparisons, as follows: (1) Primary Tumor vs Normal (PTN), and (2) CRC Metastatic in the Liver vs Normal (MLN). In order to provide a concrete PathExNET example that involves a set of significant pathways under study, we further examine how the pathways related to the primary tumor behave in the case of liver metastasis. Specifically, focusing on the pathways obtained from PTN comparison, we used PathExNET to obtain their expression status using as input the PTN and MLN expression datasets, accordingly. Table 1 shows the results of this analysis by means of the CombinedFC equation proposed in this work.

**Table 1:** Table shows how the expression status of pathways related to the primary tumor behave in the case of liver metastasis and vice-versa. The red colored values depicted with bold font, indicate an increase on the expression value.

	Pathway Names from PTN comparison	PTN dataset	MLN dataset	Pathway Names from MLN comparison	PTN dataset	MLN dataset
1	glycolysis	**1.288**	1.221	post-translational protein phosphorylation	0.982	**1.228**
2	glucose metabolism	**1.353**	1.104	phase i - functionalization of compounds	0.773	**1.103**
3	transcriptional regulation by runx3	**1.056**	0.853	transport of mature mrna derived from an intron-containing transcript	1.404	**1.434**
4	regulation of plk1 activity at g2/m transition	1.126	**1.188**	transport of mature transcript to cytoplasm	1.427	**1.454**
5	loss of nlp from mitotic centrosomes	1.270	**1.319**	eukaryotic translation elongation	1.423	**1.633**
6	recruitment of mitotic centrosome proteins and complexes	1.233	**1.295**	peptide chain elongation	1.435	**1.643**
7	loss of proteins required for interphase microtubule organization from the centrosome	1.270	**1.319**	viral mrna translation	1.435	**1.630**
8	centrosome maturation	1.233	**1.295**	eukaryotic translation termination	1.42	**1.635**
9	regulation of apc/c activators between g1/s and early anaphase	1.101	**1.370**	nonsense mediated decay	1.448	**1.649**
10	cdc20:phospho-apc/c mediated degradation of cyclin a	1.095	**1.368**	role of lat2/ntal/lab on calcium mobilization	0.835	**1.094**

**Fig. 6:**
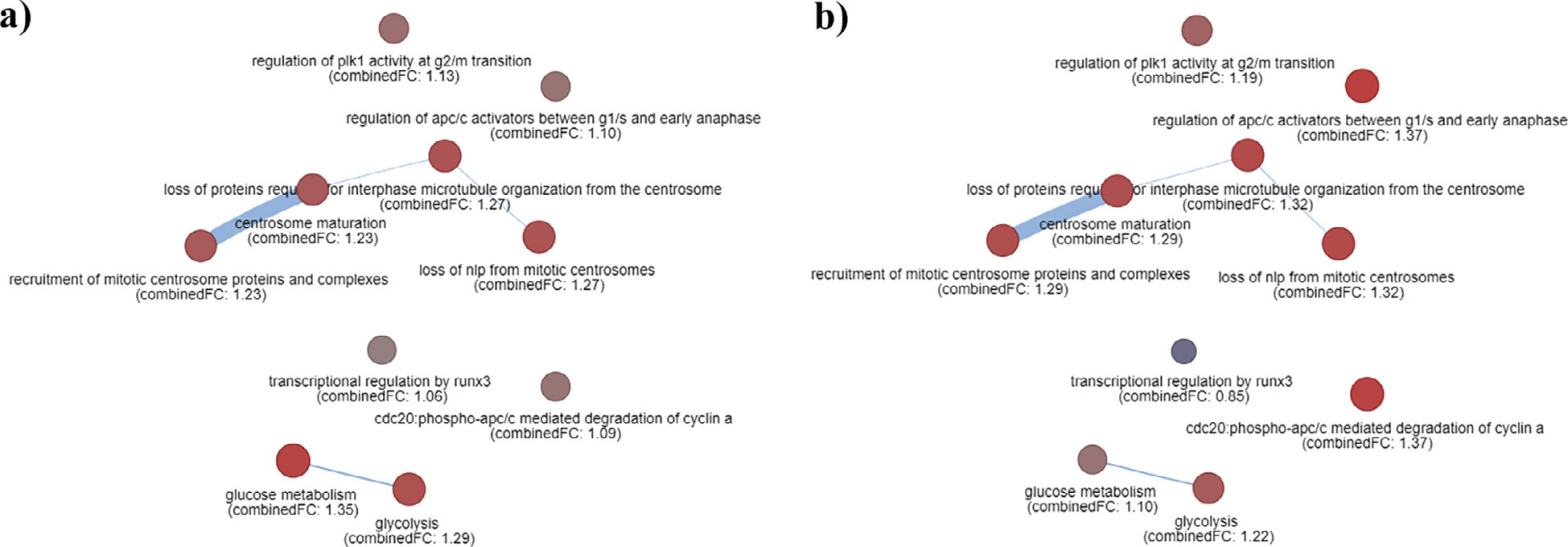
Pathway expression network showing how the expression status of pathways related to the primary CC tumor (left network), behave in the case of liver metastasis (right network). The color scale describes the color for different values of CombinedFC score.

Herein a significant observation is that the first three pathways have been found to be higher expressed in the case of primary tumor (PTN dataset), while the rest of them have been found to be higher expressed in the case of liver metastasis (MLN dataset). On the contrary, the significant pathways obtained from MLN comparison seem to remain highly expressed in liver metastasis comparing to the primary tumor. However in both lists of pathways under study, the overall pathway-to-pathway network is higher expressed in the case of liver metastasis, where the percentage increment rate $\left(R=\frac{\#ofincrements}{\mathrm{total}pathways}\times 100\right)$ was found R=70% in the case of primary tumor pathways and R=100% in the liver metastasis. We recall that the above results by no means suggest any objective truth on candidate colorectal cancer pathways, but simply demonstrate a well-designed example for understanding the significance of PathExNET tool on performing comparative analyses of pathways across different experiments. Even more, the above difference of the expression across experiments, clearly shows that the statistical significance of a pathway related to a specific disease is not necessary related to the expression status of the genes included in the pathway. Fig. 6 indicatively depicts the two identical pathway expression networks showing the difference between the expression values of primary tumor obtained pathways, using as input the PTN and the MLN datasets, accordingly.

## 4 Novelty and applications of PathExNET

Offering web-based services for pathway based analyses, is an important forward technological step that aims to reduce the complexity and expertise required for searching, obtaining and combining information from large repositories with biological content. At the same time such web-services help users to avoid a significant amount of local resources required to perform such analyses at a single computer level, as well as compatibility issues and stringent installations in some cases. In this line, PathExNET provides a freely available, well-designed and easy to use framework of analysis, to perform post experimental pathway based analysis in the context of the proposed mathematical framework. To the authors’ knowledge, there is not any available web-tool able to perform such entire workflow. PathExNET has been successfully applied in a study that has been recently published by our group [24]. Specifically two KEGG pathways were examined and evaluated, across 16 ataxia-related and 6 spasticity-related human gene expression microarray datasets, namely the “*sphingolipid signaling pathway*” and the “*Sphingolipid metabolism*” accordingly. PathExNet was used to calculate the combined fold change status for each of the two pathways, based on the expression change of the genes that participate in the respective pathways. The underlying methodology was executed for each dataset separately isolating the genes that participate in each of the selected pathways along with thelogFC and p-value metrics, as provided by the differential expression analysis. Using PathExNet, the authors concluded to a list of genes that participate in the “*sphingolipid signaling pathway*” and the “*sphingolipid metabolism”* for each dataset of each tissue. The genes that were consistently differentially expressed (over- or under-expression) across two or more datasets per tissue were further investigated in that study.

## 5 Discussion

The powerful concept of the graph theory provides significant information towards understanding the organization of entities that sustain large and complex biological systems [10], [36]. The lack of tools for pathway characterization with algorithms that do not use the typical statistical p-value estimations obtained from gene expressions, opens a relevant scientific field and interest on software development to this direction. Even more, casting biological pathways as numeric networks and analysing their topology and their properties, has become a promising and useful Systems Bioinformatics approach [35], [38], [53]. In this line, the creation of pathway expression networks proposed through the PathExNET framework seems a promising approach towards enhancing specific biological processes that may be related to specific condition under study [43]. The demonstration of the PathExNET capabilities has been based on two SARS-COV-2 datasets, available in [3], as well as on two Colorectal Cancer datasets available in [27]. Specifically, in the case of SARS-COV-2 datasets, the analysis performed by means of PathExNET concept, revealed a significant diversity of the expression status of the immune system pathways observed in-between two diverse SARS-COV-2 infected cell-lines. Analogous observation was found in the case of the two Colorectal Cancer datasets, where 3 of 10 significant primary-tumor pathways have been found to be higher expressed in the case of primary tumor, while the rest of them have been found to be higher expressed in the case of liver metastasis. Herein it should be stressed that the underlying analysis by no means suggests any objective truth related to either the SARS-COV-2 or Colorectal Cancer candidate pathways, but demonstrates a simple biological scenario that aims to show the performance and novelty of PathExNET, as a post-experimental analysis tool on gene expression datasets. On these grounds, PathExNET is expected to be a valuable tool for research on post-transcriptomic data analysis, allowing the transition from gene expression information to pathway level analysis and visualisation. PathExNET puts significant contribution to the numeric characterisation and understanding of pathway relationships, while at the same time offers a pipeline that fills a significant gap between gene expressions and pathway perturbation.

## Declaration of Competing Interest

The authors declare that they have no known competing financial interests or personal relationships that could have appeared to influence the work reported in this paper.
